# Updates on Measles Incidence and Eradication: Emphasis on the Immunological Aspects of Measles Infection

**DOI:** 10.3390/medicina58050680

**Published:** 2022-05-20

**Authors:** Ali A. Rabaan, Abbas Al Mutair, Saad Alhumaid, Mohammed Garout, Roua A. Alsubki, Fatimah S. Alshahrani, Wadha A. Alfouzan, Jeehan H. Alestad, Abdullah E. Alsaleh, Maha A. Al-Mozaini, Thoyaja Koritala, Sultan Alotaibi, Mohamad-Hani Temsah, Ali Akbar, Rafiq Ahmad, Zainab Khalid, Javed Muhammad, Naveed Ahmed

**Affiliations:** 1 Molecular Diagnostic Laboratory, Johns Hopkins Aramco Healthcare, Dhahran 31311, Saudi Arabia; 2 College of Medicine, Alfaisal University, Riyadh 11533, Saudi Arabia; 3 Department of Public Health and Nutrition, The University of Haripur, Haripur 22610, Pakistan; 4 Research Center, Almoosa Specialist Hospital, Al-Ahsa 36342, Saudi Arabia; abbas.almutair@almoosahospital.com.sa; 5 College of Nursing, Princess Norah Bint Abdulrahman University, Riyadh 11564, Saudi Arabia; 6 School of Nursing, Wollongong University, Wollongong, NSW 2522, Australia; 7 Administration of Pharmaceutical Care, Al-Ahsa Health Cluster, Ministry of Health, Al-Ahsa 31982, Saudi Arabia; saalhumaid@moh.gov.sa; 8 Department of Community Medicine and Health Care for Pilgrims, Faculty of Medicine, Umm Al-Qura University, Makkah 21955, Saudi Arabia; magarout@uqu.edu.sa; 9 Department of Clinical Laboratory Sciences, College of Applied Medical Sciences, King Saud University, Riyadh 11362, Saudi Arabia; ralsubki@ksu.edu.sa; 10 Department of Internal Medicine, College of Medicine, King Saud University, Riyadh 11362, Saudi Arabia; falshahrani1@ksu.edu.sa; 11 Department of Internal Medicine, Division of Infectious Diseases, College of Medicine, King Saud University Medical City, Riyadh 11451, Saudi Arabia; 12 Department of Microbiology, Faculty of Medicine, Kuwait University, Safat 13110, Kuwait; alfouzan.w@hsc.edu.kw; 13 Microbiology Unit, Department of Laboratories, Farwania Hospital, Farwania 85000, Kuwait; 14 Immunology and Infectious Microbiology Department, University of Glasgow, Glasgow G1 1XQ, UK; jeehanalostad@gmail.com; 15 Microbiology Department, College of Medicine, Jabriya 46300, Kuwait; 16 Core Laboratory, Johns Hopkins Aramco Healthcare, Dhahran 31311, Saudi Arabia; abdullah.e.alsaleh@gmail.com; 17 Immunocompromised Host Research Section, Department of Infection and Immunity, King Faisal Specialist Hospital and Research Centre, Riyadh 11564, Saudi Arabia; mmozaini@gmail.com; 18 Division of Hospital Internal Medicine, Mayo Clinic Health System, Mankato, MN 56001, USA; koritala.thoyaja@mayo.edu; 19 Molecular Microbiology Department, King Fahad Medical City, Riyadh 11525, Saudi Arabia; salotaibi1@gmail.com; 20 Pediatric Department, College of Medicine, King Saud University, Riyadh 11451, Saudi Arabia; mtemsah@ksu.edu.sa; 21 Department of Microbiology, University of Balochistan, Quetta 87300, Pakistan; aliakbar.uob@gmail.com; 22 Department of Microbiology, The University of Haripur, Haripur 22610, Pakistan; rafiq.ahmad@uoh.edu.pk (R.A.); zainabkhalid675@gmail.com (Z.K.); javed.muhammad@uoh.edu.pk (J.M.); 23 Department of Medical Microbiology & Parasitology, School of Medical Sciences, University Sains Malaysia, Kota Bharu 16150, Kelantan, Malaysia

**Keywords:** measles, immunity, vaccine, MCV1, immunosuppression

## Abstract

Measles is an RNA virus infectious disease mainly seen in children. Despite the availability of an effective vaccine against measles, it remains a health issue in children. Although it is a self-limiting disease, it becomes severe in undernourished and immune-compromised individuals. Measles infection is associated with secondary infections by opportunistic bacteria due to the immunosuppressive effects of the measles virus. Recent reports highlight that measles infection erases the already existing immune memory of various pathogens. This review covers the incidence, pathogenesis, measles variants, clinical presentations, secondary infections, elimination of measles virus on a global scale, and especially the immune responses related to measles infection.

## 1. Introduction

Measles is a lethal infectious disease caused by an RNA virus belonging to the paramyxoviridae family. It was reported that a single measles case can cause 12–18 secondary infections in a healthy population [1]. The measles virus is genetically related to the rinderpest virus, which causes infection in cattle. This virus co-evolved in the communities where humans and cattle lived together for a long time. This virus was declared to be eradicated by the World Organization for Animal Health in 2011 [2]. Epidemiological studies suggest that humans acquired measles infection 5000–10,000 years back due to expanding agricultural practices and the domestication of cattle for these same practices [3].

Before the development and introduction of the measles vaccine in the 1960s, it was the leading cause of child mortality and morbidity globally. Before introducing the measles vaccine, measles alone caused more than 2 million childhood deaths. The inclusion of the measles vaccine in routine immunisation programs and government-sponsored awareness programs has resulted in a drastic decline in measles-related morbidity and mortality. Despite the efforts made by the government and the widespread immunisation programs, measles results in more than 100,000 deaths annually [4]. This review highlights the incidence and epidemiology of measles, the viral pathophysiology, prevention, diagnosis, and management, progress made in the virology of this disease, measles elimination, eradication challenges, and the immunological aspects of measles infection.

### Measles Incidence

The annual incidence of measles in 2000 was 145 globally per million people, but with the rapid implementation of immunisation programs worldwide, the annual incidence declined from 83% to 25% [5]. In 2017, there were 109,000 predicted deaths from measles globally compared with 545,000 deaths due to measles in 2000. From 2000 to 2017, immunisation programs prevented approximately 21.1 million deaths worldwide [5]. After reaching a historic low of fewer than 100,000 deaths in 2016, the number of deaths due to the illness increased to over 140,000 in 2018 [6]. The number of measles cases globally increased by 167% in 2018 compared with 2016 [6].

During 2018 and 2019, there was an increase in the reported cases of measles in countries such as the United States and several other developed countries. Countries such as Madagascar, Ukraine, India, Brazil, Philippines, Venezuela, Thailand, Kazakhstan, Nigeria, and Pakistan also showed an increase in the reported incidence of measles recently [7]. As per World Health Organization (WHO) reports, most measles cases come from countries with poor healthcare systems. Vaccine refusal and hesitancy were the primary cause of the increase in the incidence of measles, which is among the top 10 global health threats in 2019 [7]. The World Health Assembly approved a global vaccine action plan in 2012 to eradicate measles, rubella, and congenital rubella syndrome in six of the World Health Organization regions by the year 2020. However, the plan did not meet its intended targets, and a strategic framework was designed to eliminate both measles and rubella by 2030 [8]. 

A few studies reported the incidence of measles in Saudi Arabia. As per one report in 2015, there was an increase in the measles incidence per 1,000,000 from 3.2 in 2009 to 12.8 in 2011. The incidence reduced to 9.9 in 2012 [9]. The majority (50%) of measles-infected cases were in children younger than 5 years old. About 39% of the measles-infected children were unvaccinated [10]. The spike in cases and deaths globally due to measles is alarming. The emergence of the COVID-19 pandemic can potentially lead to a further spike in measles cases and deaths due to the disruption of immunisation events and activities worldwide [7].

## 2. Measles Virus: Transmission and Infectious Cycle

Respiratory droplets and small aerosol particles transmit the measles virus. The virus has a high affinity for lymphocytes, and it was shown in transgenic mouse models that the virus infects the alveolar macrophages and dendritic cells of the lungs in just 2 days [11,12]. The infection starts in the lower respiratory tract before it progresses towards the upper respiratory tract. The virus also spreads to the skin, conjunctiva, and other organs. These particles remain suspended for 2 h [13]. The measles virus has an incubation period of 10 to 14 days. Infected patients exhibit coryza, cough, and conjunctivitis at the end of the prodromal phase. Koplik spots in the cheeks follow this, and a rash spreads from the face to the toes [12]. The onset of fever occurs around 10 days after infection and usually lasts for 7 days [12]. A recent systematic review reported a median incubation period of 12.5 days for the measles virus with a range from 11.8 to 13.2 days. Lessler et al. (2009) [14] indicated that the infectious cycle begins days before the appearance of a rash. The appearance of the rash is accompanied by viremia, cough, and coryza. The intense cough and coryza facilitate viral transmission. It is difficult to set a specific infectious period for the measles virus, with reports suggesting the presence of viral RNA in the blood, urine, and nasopharyngeal specimens several months after the onset of a rash [15]. Although the general population has access to an efficient vaccine for measles, it remains one of the most infectious diseases, with a basic reproductive number (R_0_) of 12–18 [16]. The R_0_ represents the contagiousness of the measles virus. R_0_ is the average number of secondary cases of measles infection caused by the introduction of an infected person into a susceptible population [17]. In the case of measles, an R_0_ of 12–18 means that an infected patient with measles can infect anywhere between 12 and 18 healthy individuals. R_0_ depends on several factors, such as transmission pattern, population density, and social contact habits, and is reported to be 9–18 in different circumstances [1]. The R_0_ for measles is higher than the contagious viral infections, such as smallpox (R_0_ = 5–7) and influenza (R_0_ = 2–3) [7]. Such a high value of R0 represents the measles virus’s highly contagious nature, which poses a major obstacle to its elimination. Due to such high transmissibility levels, vaccination rates ranging between 89 and 94% must be required to eliminate measles and encourage herd immunity in a susceptible population [18]. The measles virus has a high propensity to stay in the population and cause secondary infections through unbroken chains of transmission. Since the measles virus does not cause persistent or latent infections and does not have animal reservoirs, eradicating measles is quite possible [19]. The measles virus can also infect non-human primates, but due to their low community size (300,000 to 500,000), the viral transmission cannot be sustained [20].

Endemic measles virus transmission shows a specific seasonal pattern [11]. Measles outbreaks happen mostly during late winter and early spring. The seasonal outbreak is supported by social contact patterns, such as increased individual contact between school children and the typical environmental factors favouring viral transmission in temperate climates [1]. Measles outbreaks are more variable in tropical regions [21]. During the initial months after birth, infants are protected from measles infection by the maternal anti-measles virus IgG antibodies that are received passively from the mother [13]. The infants born to women with naturally acquired immunity against wild-type measles virus infection are less susceptible to measles infection than infants born to women with vaccine-induced immunity due to higher titres of anti-measles IgG in women infected with the wild-type measles virus [22,23].

The average age of measles infection depends on factors such as the decline in maternal immunity, contact patterns, and age of acquired immunity against measles infection. With an increase in the vaccine coverage and prevention of contact between infected persons and the healthy population, the age of measles infection has shifted from infancy to adolescence and adulthood [24]. 

## 3. Measles Virus: Structural and Functional Aspects

The measles virus belongs to the genus Morbillivirus. It is a member of the Paramyxovirinae subfamily and the family Paramyxoviridae. It is an enveloped, single-strand, non-segmented, negative-sense RNA virus. The virus manufactures pleiomorphic viral particles ranging from 150 nm to 900 nm [17]. Like other viruses belonging to the morbilliviruses, the measles virus is highly contagious and is spread via the respiratory route [25].

The viral RNA genome is 15,894 nucleotides long and encodes for six structural proteins and two non-structural proteins. The structural proteins are the nucleocapsid protein (N), the matrix protein (M), the phosphoprotein (P), the haemagglutinin protein (H), the fusion protein (F), and the polymerase protein (L). The two non-structural proteins are V and C, encoded by the P gene, which is instrumental in modulating the cellular response to infection [26,27]. While the virus exits the host cell, the virus takes up the cell membrane of the host cell, forming the virus’s envelope. This envelope acquired from the host cell helps the virus evade the host cell’s immunity. Interior to the envelope is the matrix made up of a protein called M. The genomic RNA is surrounded by a helical nucleocapsid protein (N). Special proteins L and the phosphoprotein P help in the replication of the viral genome and making copies of the virus. The haemagglutinin (H) and fusion (F) glycoproteins form the surface projections on the envelope. These two glycoproteins interact with or without additional cellular proteins (receptors) that help the virus bind and enter into immunological cells, such as lymphocytes, monocytes, macrophages, dendritic cells, and the epithelial cell adherens junctions [26]. These proteins determine the viral specificity for different cell types and tissues. Infection with measles imparts life-long immunity against the virus due to the production of IgG antibodies and memory cells against the haemagglutinin protein that blocks the binding of the virus to the host cell receptors [28].

On the other hand, the fusion protein helps the virus enter the host cell by enabling the fusion of the viral envelope to the host cell membrane [29]. Based on the sequence of the variable region of the nucleoprotein, 24 different strains of the measles virus have been identified by the WHO [30]. Many of these measles virus genotypes are no longer detected in the infected population [31,32]. Despite the genetic diversity, the measles virus is antigenically monotypic. An attenuated measles vaccine derived from the Schwarz and Moraten measles vaccine strains that belong to a single genotype is still protective against measles [12]. The measles virus is an RNA virus with a high mutation rate, giving rise to evolving viral strains. Since the vaccines for the measles virus are directed against the highly conserved epitopes of the haemagglutinin protein, there is no need to develop vaccines against the new evolving strains of the measles virus [33].

### 3.1. Pathophysiology

The measles virus is transmitted through respiratory droplets or aerosol particles. The initial site of infection is the respiratory tract, where they infect the macrophages in the lung alveoli, lymphocytes, and dendritic cells [12,34]. The virus proliferates during the incubation period and migrates to the lymphoid tissues, and then gets spread to the bloodstream by the infected lymphocytes, endothelial cells, and epithelial cells [35]. Wild-type measles vectors are known to infect cells by using the nectin-4 receptors and signalling lymphocytic activation molecule 1 (SLAMF1, also known as CD150) [26,27,36]. The viral strains are also known for their affinity towards the CD46 receptor molecule expressed in human cell membranes [37]. The measles virus can enter through a pH-independent pathway initiated by endocytosis and facilitated by SLAMF1 in B-lymphoblastoid cells [38,39]. In addition, the virus was shown to enter breast and cancer cells (MCF7) through the micropinocytosis pathway mediated by the nectin-4 receptors [25]. Nectin-4 receptors that are present on the surface of the epithelial cells in the respiratory tract help the virus transmission from the infected dendritic cells to the epithelial cells of the respiratory tract [40]. The measles virus is transmitted to the susceptible hosts either through budding from the surface of epithelial cells of the respiratory tract or through the damaged epithelial cells [25]. The measles infection starts before the appearance of a rash, which is typical of a measles infection, and lasts for several days after the appearance of the rash. The viral RNA can be detected for at least 3 months in clinical samples after the appearance of the rash [15]. Measles infection is traditionally believed to be an acute infection that lasts for 2–3 weeks. Still, recent studies on the macaque model reported the presence of viral RNA in the peripheral mononuclear cells for 67 days [41]. According to this study, the measles viral RNA remains detectable in the lymphoid tissues even after blood samples disappear [41].

### 3.2. Clinical Variants of Measles

There are two clinical variants of measles infection: modified measles and atypical measles. 

#### 3.2.1. Modified Measles

Modified measles is a milder form of measles that is reported in patients with a history of either wild-type measles infection or vaccination. Modified measles have a longer incubation period, ranging from 17 to 21 days [42]. Modified measles is not highly contagious [43]. Several causes for the inefficient/non-protective immunity for the measles virus result in modified measles. Transplacental acquisition of measles-specific antibodies from mother to infant is one of the reasons for modified measles in infants. The measles-specific antibodies acquired from infected mothers are generally cleared from the bloodstream of infants by 3–9 months of infancy [22]. When the antibody titres reach sub-optimal levels for protection against measles, infants become susceptible to measles infection. Still, the disease is milder due to low levels of protective antibodies. Other causes of modified measles are intravenous antibodies, measles vaccination resulting in lower antibodies than required for measles protection, and a prior history [3].

#### 3.2.2. Atypical Measles

Atypical measles is now rare in individuals vaccinated with the inactivated measles virus. This form of measles vaccine was used back in 1963 and 1967 in the United States. The inactivated virus vaccine could not produce protective levels of measles-specific antibodies [44]. Symptoms like headache and fever appear within 7 to 14 days of exposure to inactivated measles virus vaccine [17]. Unlike the typical measles infection, atypical measles is associated with the appearance of the rash first in the extremities and then extends to the trunk [45,46]. Atypical measles results in severe infection, but it is not contagious [47]. 

## 4. Immunological Aspects of Measles Infection and Host Immunity

The measles virus is known to infect different cell types. However, most of the studies that were performed on the virus were performed on tumor cell lines in vitro. A few studies were performed on IFNAR knockout mice and Rhesus monkeys [48]. These in vitro and in vivo studies have allowed us to understand the immunological aspects of the disease and the host’s immune responses to it. 

### 4.1. Innate Immunity Response to Measles Infection

The measles virus spreads through its mobility, starting from the site of infection to the respiratory tract, lymphoid organs, and then back to the respiratory tract. It first infects CD150-expressing cells, namely, the dendritic cells (D.C.s) and macrophages (M.P) (Lemon et al., 2011). D.C.s play a central role in developing an immunological response to pathogens by presenting antigens on major histocompatibility complex molecules (MHC), T-cell activation due to costimulation of CD80, and CD86, which ultimately leads to the release of proinflammatory cytokines IL-12 and IL-18. The production of IL-12 by D.C.s is important for the optimal activation of CD4+ and CD8+ T cells. The activation of the CD4+ and CD8+ cells is initiated by interferon-gamma (IFNγ). This is crucial as it activates the innate immune response to infection and reactivates during the pathogen reencounter [7]. 

### 4.2. Overwhelming of Innate Immune Response by Measles

The measles virus can block the production of IL-12 by using the Fc-gamma receptor pathway [49]. The Fc-gamma receptor (FcγR) belongs to the Fc portion of IgG, and it is heavily involved in modulating immune responses [37]. This mechanism is thought to be involved with the interaction of the viral N protein with the FcγR molecules on the surface of D.C.s, ultimately leading to lower production of IL-12 [49]. The exact mechanism of how it happens is not yet that clear. Alternatively, the complement regulator protein CD46, in conjunction with the H glycoprotein of the virus, is known to downregulate the IL-12 secretion further [49,50].

Reducing IL-12 levels after any active measles infection prevents T cell activation and differentiation, which lowers immunity. The H protein, especially in wild strains, can also trigger the activation of Toll-like receptors (TLR1, TLR2, and TLR6) and myeloid differentiation protein 88 (MyD88) on the D.C.s and MP [51]. This might lead to the activation of nuclear-factor-activated protein (NF-kB), which can regulate the expression of CD150, along with other cell surface receptors. CD150 upregulation might increase infection in cells exposed to the measles virus [52]. This can also contribute to the development of MeV infection. NF-kB is also involved in the expression of inflammatory cytokines (IL-12, IL-6, IL-18, IL-1β), chemokines (C-X-C motif chemokine ligand 8 or CXCL-8), and tumour necrosis factor alpha (TNFα) [53]. These interplay mechanisms highlight the role of D.C.s in the measles-induced immunosuppression early on in the infection stages. A lack of optimal T cell activation can generate suboptimal T cells that are instrumental in the upregulation of CD150, allowing for measles virus entry, migration to the lungs, and propagation in the host cells [54]. Neutrophils and granulocytes, among other cells, respond to the viral infection by producing IL-12 and will skew the T cell responses [55]. This could also cause a reduction in the interactions with the major histocompatibility complex molecules (MHC) and the stimulation of T cells.

The early stages of infection with wild-type measles virus are highlighted by secretions of TNF and IFNγ [56]. Elevated levels of IL-4 and IL-10 cytokines are seen in the later stages of infection [57,58], and they could lead to immunosuppression [59]. The measles virus causes a loss of memory B and naïve cells and delays reconstitution of these B cells, leading to immune amnesia [60]. Although the loss of B cells and the infection contribute significantly to immune amnesia, the interactions between the measles virus and D.C.s also contribute to lower immunity. D.C.s’ role in B cell education is a well-known fact [61]. They are involved in activating the CD4+ T cells and then guide them through their differentiation into T follicular regulatory (Tfr) or T follicular helper (Tfh) cells. The Tfh cells, in conjunction with follicular D.C.s (FDCs), contribute towards B cell selection in the germinal centres of lymph nodes, leading to the formation of long-lived plasma cells (LLPC), which are crucial in the production of antibodies. Although the host immunity is initially severely affected by the measles virus, the infection surely produces anti-measles immune responses quickly [25,62]. The lymphopenia produced by the measles virus is short-lived and quickly replaced by lymphocytes specific to the measles virus. This clearly shows us that the D.C. functions are momentarily blocked. The measles virus uses this temporary D.C. blockade to efficiently enter, replicate, and amplify in the primed immune cells. D.C. generation and its effects on the response of B cells and T cells are dependent on the type-1 IFN system [63]. This system is crucial in activating the important cytokines for innate and adaptive immunity.

### 4.3. Innate Immunity Responses at the Cellular Level to the Measles Virus

The type-I IFN system is a cell-intrinsic immune response that consists of multiple pathways that lead to upregulation of the IFN proteins (IFNα or IFNβ) and subsequent activation of the Janus kinases, signal transducer, and activator of transcription proteins (JAK-STAT) pathway [64]. IFNγ1, -2, and -3, which constitutes the type-III IFN system, also detect the measles virus [65]. Even though their signals are sent through a different receptor, the downstream signalling pathway is identical to that of the type-I IFN system [66]. Thus, both type-I and type-III systems work in conjunction to activate the innate immune responses in the mucosal tissues. The measles virus, which is a double-stranded RNA (dsRNA), is detected through various cytoplasmic RLRs (retinoic-acid-inducible gene-1-like receptors) that include RIG-I, laboratory of genetics and physiology (LGP2), and melanoma differentiation gene-5 (MDA-5) [67,68]. Activating the MDA5/RIG-I receptors leads to the binding to mitochondrial anti-viral signalling adapter (MAVS). This binding leads to an activation cascade involving TANK-binding kinase 1 (TBK1), interferon regulatory factor 3 (IRF3), and NF-kB [66]. Transcription factors of NF-kB and IRF3 play an essential role in the expression of IFNβ. TLR3 and TLR7 play an important role in producing type-1 IFN as soon as they detect the dsRNA or ssRNA in the endosomes [69]. IFNα is expressed via the activation of the IRF7 via the TLR7 signalling pathway, which depends on the MyD88/IKKα [69]. IFNα and IFNβ attachment to the type-1 IFN receptor (IFNAR) results in an IFN signalling cascade. The signalling cascade further activates the Janus kinases (JAK1, tyrosine protein kinase (TYK2)) and phosphorylation of STAT1 and STAT2 proteins. Janus kinases and STAT proteins then combine with interferon regulatory factor 9 (IRF9) to form an interferon-stimulated growth factor 3 (ISGF3), which then helps in the expression of interferon-stimulated genes (ISGs) [70]. These ISGs are crucial for innate immune sensing. They can either exhibit direct anti-viral responses through interferon-inducible inhibitory protein (viperin or RSAD2) leading to restriction of measles virus release [71] or regulate enzymes like adenosine deaminase acting on RNA (ADAR1), which form an integral part of the innate immune sensing pathways [72,73].

### 4.4. Measles Virus Evading the Host’s Immunity Mechanisms

The measles virus has evolved an extensive immune evasion mechanism. It interferes with IFN responses, making the IFN system the most important innate and adaptive immunity [48]. The measles virus belongs to the genus *Morbillivirus* of the *Paramyxoviridae* family [39]. The genome of the measles virus encodes for eight viral proteins N-P/V/C-M-F-H-L [74]. The measles virus genes attack all the parts of the IFN system right from their induction to their signalling cascade. The P/V/C gene in the measles virus expresses proteins P, V, and C, which form the main antagonists to the IFN system. The V protein mainly targets cellular proteins [59]. The V protein is characterised by the amino n terminal and carboxy-terminal domains [48,75] The carboxy-terminal of the V protein binds tightly to the STAT2 protein, thereby inhibiting the IFN signalling pathway [64,76,77].

On the other hand, the amino-terminal domain of the V and P proteins binds with STAT1 [78,79]. However, the binding needs the presence of STAT2 to make it efficient [77]. The phosphorylation of STATs is also blocked by the V protein [80]. In short, the measles V protein blocks the STAT activation. The V protein also targets MDA-5 through the charged residues on the carboxy-terminal of the V protein [81]. V protein also targets RIG-I and LGP2 receptors, effectively blocking any chances of RLR activation [82]. Finally, the V protein also blocks the phosphorylation of IRF7 by acting as a substrate for IKKα and, therefore, prevents the attachment of IRF7 to the IKKα [74]. The ability of the measles virus protein to block IKKα/IF7 is advantageous due to its ability to infect D.C.s via the prevention of any TLR7 mediated production of IFN in plasmacytoid DC [59]. The C protein of the P/V/C is thought to alter the viral replication by regulating the RNA replication, which ultimately leads to lower production of defective interfering RNA (DI RNA) [83,84]. The reduced production of DI RNA is beneficial for the virus, as it was shown to activate both RIG-I and MDA-5 [68,85]. The measles virus also relies on a cell-intrinsic immune system to suppress its innate immune response. The ADAR1 protein does that by preventing the activation of the dsRNA response [86].

### 4.5. Infection and Host Immunity 

Host immune response is responsible for the neutralisation of the virus and the establishment of immunity for further infections [78]. The viral proteins V and C inhibit the expression of interferons in the host cells facilitating the replication and spread of the measles virus [83]. It is followed by the action of cellular and humoral immunity. 

Adaptive immunity is responsible for the recovery and establishment of long-term protection against measles virus infection. The first antibodies formed against the measles virus are the IgMs formed at the time of rash. IgMs stay in the bloodstream for 6–8 weeks. Detection of IgM in the bloodstream by enzyme-linked immunosorbent assays (ELISA) is used to confirm measles virus infection [87]. Subsequently, IgG antibodies against the measles virus are formed. Among the IgGs formed, the most abundant IgGs are formed against the viral nucleoprotein [88]. Cellular immunity is essential for viral clearance and recovery. Studies have shown that children with agammaglobulinemia recover from measles, but those with T-cell deficiencies do not recover from the infection and develop severe infections leading to death [85]. Several other studies conducted on macaque models of infection have further shown the role of cellular immunity in viral clearance and recovery [89]. During acute stages of infection, Th1 mediated immune response is most active, and the levels of interferon-γ levels increase [56]. During the recuperation phase, the Th2-mediated immune response is predominantly marked by the prevalence of antibodies against the measles virus. The Th2-mediated immune response is also marked by an abundance of interleukin 4, interleukin 10, and interleukin 13 [90]. 

Weak or deficient innate and adaptive immune responses can result in severe measles infections and sequelae of events leading to secondary infections. Measles infection is the first immunosuppressive viral infection described [62]. Transient lymphopenia occurs during measles infection due to the migration of the lymphocytes from the bloodstream to the lymphatic tissues, the primary sites of measles virus proliferation [25,91,92]. Studies showed abnormal cellular immunity during measles infection, such as a declined lymphocyte proliferation and an inhibited function of dendritic cells [93]. These studies were conducted in ex vivo conditions. Therefore, it is not clear whether these immunosuppressive activities of the measles virus infection also apply in the in vivo conditions [94]. 

Increased interleukin 10 was reported to remain in the blood plasma of infected children for weeks. The elevated interleukin 10 might result in immune suppression [54]. Recent studies showed that measles infection erases the immunological memory against several antigens in the host, resulting in immune amnesia. Immune amnesia results because of replacing the pre-existing memory cells with measles-virus-specific lymphocytes and may lead to increased susceptibility to previously encountered pathogens [94,95]. This immune suppression is due to a measles virus infection that may last several weeks [96]. 

## 5. Diagnosis 

Measles is easily identified by clinical features, such as fever and rashes, especially during outbreaks or in patients travelling from measles-endemic areas. Some other viral infections, such as those caused by the rubella virus, human herpesvirus type 6, parvovirus B19, and dengue viruses, show similar clinical features [19,97].

While diagnosing a suspected measles patient, clinicians should consider the typical clinical features of measles infection and look for the secondary problems due to measles infection, such as pneumonia, conjunctivitis, otitis media, and diarrhea. Since malnutrition, especially vitamin A deficiency, immune deficiency (HIV infection), and immunosuppression (individuals undergoing organ transplantation), are major contributors to measles-associated mortality, a thorough clinical examination and a detailed history of patients should be taken. Patients with these risk factors are at a higher risk of mortality. Prompt action by clinicians is warranted in patients with vitamin deficiency, which involves administering vitamin A supplements to these patients. The hospital administration should take appropriate action to isolate the measles-infected cases to prevent the transmission of the measles virus to healthy individuals [98]. It is challenging to clinically diagnose measles infection before the appearance of the rash and in immunocompromised children who might not have the rash. Furthermore, it is challenging to diagnose measles infection in individuals who had acquired antibodies against measles from the maternal immune system or through previous vaccination, in cases of mild illness, and in cases with less evidence of a rash [68].

The most widely employed laboratory test detects IgM specific for measles virus antigens by ELISA. The levels of measles-virus-specific IgM are very low or undetectable till 4 days after a rash appears. Therefore, tests within 4 days of rash development give false-negative results [45]. Almost all the measles-infected individuals show detectable levels of measles virus-specific IgM after 4 days of rash appearance, and 75% of measles-virus-infected individuals show detectable levels of measles-virus-specific IgM within the first 3 days of rash appearance. IgM levels specific to the measles virus are highest during 1–3 weeks post rash onset and decline to undetectable levels within 4–8 weeks post rash onset [5]. 

Although the commercially available ELISA kits detect antibodies for the measles virus, the standard gold method for diagnosing measles infection is the highly sensitive plaque reduction neutralisation assay [99]. Studies showed that individuals who tested seronegative for measles using ELISA were positive for seroconversion when using the plaque reduction neutralisation assay [99]. Therefore, the individuals who test negative with ELISA should be advised for plaque reduction neutralisation assay. DNA-based methods, such as real-time PCR (RT-PCR), are used to confirm a measles virus infection. RT-PCR detects the viral RNA in clinical samples of the suspected individuals even before the measles-specific antibodies reach detectable levels. Different specimens are used to detect measles-specific antibodies and viral RNA, such as oral fluids and serum extracted from dried blood spots. Although oral fluid samples are appropriate for large-scale survey purposes, this method has a low sensitivity [100,101].

## 6. Clinical Presentation

Measles is a self-limiting acute contagious viral infection. It is characterised by a typical rash, high fever (up to 104 °F), cough, coryza, and conjunctivitis. Characteristic white lesions called Koplik’s spots form in the buccal mucosa even 1–2 days before the appearance of the rash; therefore, Koplik’s spots can indicate measles infection, even before the appearance of the rash. A flat red rash appears first on the face and the neck and subsequently advances to the trunk and the extremities in solid and discrete spots within 3–4 days after the onset of fever. Children with previous immunisation history present minimal rash and sometimes do not show the typical characteristics of measles infection, i.e., cough, coryza, and conjunctivitis [102]. Malnourished children present with a prominent, deeply pigmented rash [25]. As a rash is a result of innate/cell-mediated immunity, in conditions of compromised cellular immunity, e.g., HIV, a measles infection may not develop a rash, or the appearance of the rash may be delayed. Generally, in uncomplicated cases of measles infection, the recovery takes place within 1 week of rash appearance [103]. Several factors complicate measles infection, affecting most organs. The factors contributing to the complication of measles are young age (infants), age older than 20 years, pregnancy, malnourishment, vitamin A deficiency, and immunocompromised conditions [104].

### Secondary Complications

Owing to its immunosuppressive effects, measles infection results in several secondary infections by opportunistic bacteria and viruses. The respiratory tract is the primary system affected by secondary infections following a bout of measles infection per se. Measles infection per se can cause Hecht’s giant cell pneumonia [82]. Pneumonia can also result from secondary bacterial and virus infections, accounting for a significant proportion of measles-associated mortality. The other most frequent respiratory complications associated with measles infection are laryngotracheobronchitis (croup) and otitis media. Bacterial and protozoan infections secondary to measles infection also led to diarrhoea, which is a major contributor to the morbidity and mortality associated with measles infection [80]. 

Kerato conjunctivitis was a leading cause of blindness due to measles infection. The measles vaccine and vitamin A supplementation have decreased the incidence of blindness caused due to kerato conjunctivitis from measles infection. Measles during pregnancy may result in low birth weight, spontaneous pregnancy loss, and maternal and prenatal mortality [105].

The measles virus also infects the central nervous system (CNS). It results in three rare conditions in the CNS, viz., acute disseminated encephalomyelitis (ADEM), measles inclusion body encephalitis (MIBE), and subacute sclerosing panencephalitis (SSPE). The major clinical presentations of ADEM are fever and seizures. It is a demyelinating autoimmune disease [17]. The incidence of ADEM is 1 in 1000 individuals infected with measles and can develop within days to weeks of infection. MIBE is an active brain infection resulting in neurodegeneration and mortality in individuals with compromised cellular immunity, such as children who received organ transplants and HIV-infected patients [106]. SSPE is caused by the response of the host immune system to the mutated virions. SSPE is a rare condition with an incidence of 1:10,000 to 1:100,000. It is a delayed response to a measles infection that appears anywhere between 5–10 years after an acute measles infection [17]. The major clinical presentations of SSPE are seizures and a gradual decline in cognitive and motor function, resulting in mortality. SSPE is seen in individuals infected with the measles virus in the first 2 years after birth [17]. A recent study documented a higher incidence of SSPE in the USA. This study showed higher incidence rates of SSPE in children who had a measles infection before they were 1 year old (1:609) as compared with children who had a measles infection before 5 years of age (1:1367) [107].

#### Management Strategies for Secondary Outcomes

There are no recommended prophylactic antibiotics available to prevent measles complications, but antibiotics can be administered in cases with secondary bacterial infections [108,109]. The measles virus itself has no particular antiviral therapy. Supportive care that provides sufficient nutrition, enough fluid intake, and treatment of dehydration with a WHO-recommended oral rehydration solution may minimise severe complications from measles [110]. This solution could replace the loss of fluids and other vital nutrients due to diarrhoea or vomiting. Eye and ear infections, as well as pneumonia, should be treated with antibiotics [111].

## 7. Management

Measles is a self-limiting viral infection. The management regime involves therapies to control/manage the associated risk factors and complications, such as dehydration, malnutrition, or nutritional deficiencies. The management of measles depends on prompt diagnosis, treatment of bacterial and viral infections secondary to measles infection, and vitamin A supplementation [100]. The United States of America was the first country in the world to abolish endemic transmission of the measles virus in late September 2016; this was also achieved in Venezuela in 2018 and in Brazil in 2019 [111]. Several other countries have confirmed measles eradication and countries from all six WHO regions have set measles eradication targets [110]. In 2019, a total of 83 countries were certified as having achieved or maintained measles elimination [111]. During the COVID-19 pandemic, there was a significant drop in measles cases worldwide. A few cases of measles have been documented in the European Union countries, including in areas where endemic transmission had previously been abolished or endemic [112]. The number of cases registered on the European continent has dropped from a few hundred each month to sporadic occurrences. No additional fatalities were reported by European Union countries in 2021 [111]. 

Vitamin A supplementation can be given to measles patients in order to reduce the measles-associated complications and mortality rates [46]. Vitamin A deficiency impacts the severity of measles since it slows recovery; it may cause measles-related problems, such as blindness; and may be linked to a higher death rate [113]. The American Academy of Pediatrics (AAP) and the WHO advocated for vitamin A treatment for hospitalised children with measles [46]. Compared with a single dose, two doses of vitamin A supplements have been shown to reduce the risk of measles-associated mortality in children younger than 2 years [30]. Vitamin A supplements should be given in two doses every 24 h to all children diagnosed with measles. This medication may help to avoid eye damage and blindness by restoring low vitamin A levels during measles infection, which can occur even in well-nourished children. Supplementing with vitamin A was also demonstrated to minimise measles fatalities [46]. However, recent studies suggested that vitamin A was not utilised correctly to treat US children with measles, either because it was not administered at all or because it was taken at too low levels [6,46].

Because measles has a long incubation period before viremia appears, there is a large window for antiviral therapy. Rapid antiviral therapy, in particular, might completely prevent infection onset in naive patients. However, since a clinical condition follows the peak of viral multiplication, protracted incubation periods may limit therapeutic potential. Although there is no effective and specific anti-viral therapy for the measles virus, some studies have shown promising results with anti-viral agents, such as ribavirin and interferon alfa in severe measles cases [65,114]. A recent study showed that ribavirin nanoparticles have significantly higher anti-viral activity than ribavirin alone [115].

### 7.1. Advances in the Administration of the Measles Vaccine

Currently, none of the WHO’s elimination goals for measles are being met. Despite this, efforts to improve the situation are still ongoing [6,116]. Currently, the measles vaccine is administered as a trivalent vaccine (MMR), along with mumps and rubella. Most of the reasons for the difficulty of eliminating the measles virus are focused on vaccinations. The problems with low coverage of vaccinations start with the vaccine in itself: the price of the vaccine, use of multi-dosage vials leading to loss of vaccine, improper reconstitution of vaccine, the lack of cold storage, and finally, the lack of expert healthcare workers [117]. To overcome such limitations, extensive research was done to formulate the vaccine as microneedle patches [118,119]. These patches are designed to be placed on the skin to administer the measles and rubella vaccine through a fine array of micron-scale needles. These micron-scale needles contain the active ingredient of the measles and rubella vaccine and other water-soluble excipients and are designed to dissolve when placed on the skin. These patches were shown to maintain their potency, even at elevated temperatures, and can be safely used in extreme temperature conditions, such as in Africa. These patches would not require any experienced healthcare workers and can be safely administered by parents. The neutralisation titters produced with the microneedle vaccine patch and the subcutaneous injections of the vaccine were the same when administered in cotton rats [117]. Similar results were obtained when the vaccine was administered as a patch or through subcutaneous injections in Rhesus monkeys [120]. While these pre-clinical studies show excellent results, there is a need for further clinical studies to see whether they exhibit similar results in human beings.

#### 7.1.1. Measles Vaccination Schedule

The Centres for Disease Control and Prevention (CDC) recommends children of age 12–15 months have the first shot of the MMR vaccine, followed by the second shot at the age of 4–6 years old [120]. However, the WHO recommended measles vaccinations in infants as early as 6 to 9 months in areas with measles outbreaks. A systematic review on early measles vaccinations at 6–9 months found that the vaccinations are safe and produce an excellent immune response. Still, the seroconversion and protection were lower when compared with infants who received the measles vaccine at older ages [121]. The age of the vaccinated infants was the most important factor that determined the vaccine’s efficacy [122]. Children with vaccinations later than 15 months were at lower risk of measles infections than children who got vaccinated between 12 and 14 months [123]. Further studies need to be performed to ascertain the best age for the first dose to be administered to infants.

#### 7.1.2. Laboratory Diagnostic Possibilities for Measuring the Effectiveness of Measles Vaccination

The measles vaccination induces both neutralising and non-neutralising antibodies against several measles proteins, as well as measles-specific cellular immunity, with a very limited association between humoral and cellular immunity measurements [121]. In specialised healthcare laboratories, the functional measles-specific neutralising antibodies (anti-H and anti-F) can be measured after a vaccination using a conventional plaque reduction neutralisation test (PRN) or the plaque reduction microneutralisation (PRMN) assay [122]. The acceptable PRN titre that neutralises antibodies for measles protection is >120 mIU/mL. However, people with low/undetectable PRN antibody levels were also shown to be protected against clinical measles, indicating that cellular immunity plays a role in protection. Apart from neutralising antibodies, serum antibodies were also demonstrated to be adequate for measles protection on several occasions [121].

Apart from the PRN or PRMN assays for the possibilities for measuring the effectiveness of measles vaccination, many seroprevalence assays are available that can measure the level of immune response due to vaccination [122]. These assays include many automated and semi-automated commercially available immunoassays, for example, microtitre-plate enzyme-linked immunoassays (EIA) and multiplex microsphere/bead fluorescence-based immunoassays, which report the quantitative or qualitative results [121].

## 8. Eradication: Goals and Progress

The World Health Assembly (WHA) 2010 set three objectives for worldwide measles control by 2015. The first objective was to increase the measles-containing vaccine coverage (MCV1) in children aged 1 year to ≥90% and ≥80% at national and district levels, respectively. The second objective was to decrease the global annual measles incidence to <5 cases per 1 million individuals. The third objective was to reduce the measles mortality rate by 95% from the 2000 estimate [124]. A recent study reported the progress towards measles elimination worldwide [121]. The MCV1 vaccine coverage increased globally from 72% to 86% during 2000–2018 (Table 1), the measles incidence declined from 145 to 49 cases per 1 million individuals, and there was a 73% decrease in the annual measles-associated mortality [5]. 

Although there was a significant decline in the overall measles-related mortality during 2000–2018, there was a global increase (about 167%) in the incidence of measles in 2018 as compared with 2016. Furthermore, measles-related mortality has increased since 2017 [6]. This measles-related mortality can be further magnified because of the current COVID pandemic. Ever since the COVID pandemic struck across the world, the WHO recommended temporarily suspending mass vaccination programs worldwide [112]. This led to lower vaccination rates, and it could be further reduced moving on forward. Due to the COVID pandemic, social distancing, mask mandates, and online education might have contributed to no measles outbreaks. However, there might be a return of measles outbreaks when people start travelling to measles-endemic areas or citizens from these areas travel around the world. A single dose of the Schwartz vaccine was started in 1982 in Saudi Arabia. It took almost a decade to introduce the two-dose measles vaccine in Saudi Arabia. A two-dose Edmonston–Zagreb measles vaccine was introduced in 1991 [125]. The first dose was given at 6 months of age and the second dose at 12 months of age. Implementing a two-dose vaccine shifted the infection age from younger to older age. Moreover, it was observed that even in the vaccinated population, about 50% of measles cases were observed in the 1–4-year-old age group [125]. 

The availability of effective vaccines for measles and efforts of the government to implement routine vaccination has reduced the measles incidence worldwide, and measles is now eradicated in some countries. Despite the preventive efforts of the government, measles outbreaks are seen in Saudi Arabia. A measles outbreak occurred in Qassim, Saudi Arabia, in 2007. About 40% of the cases were under 4 years [126]. Another outbreak was reported in Tabuk, Saudi Arabia. In this outbreak, around 46% of the suspected cases were IgM-positive for measles infection, and the majority were males from urban [108]. A recent outbreak occurred in Jeddah city, Saudi Arabia, in 2018 [113]. These outbreaks raise concern for measles eradication in Saudi Arabia and on a global scale. These reports warrant increased awareness among communities, strict implementation of vaccination programs, and periodic surveillance.

## 9. Conclusions

The countries claiming complete eradication of measles are still at risk for measles outbreaks until the virus is circulating in other populations. The measles virus can be acquired through travel in these countries. Measles outbreaks result in a financial burden on the economy. Although vaccine coverage has increased drastically in the last decade through awareness and surveillance worldwide, vaccine denial remains the major problem in eradicating measles. Since measles is associated with sequelae of secondary infections, adequate supportive therapy, such as vitamin A supplementation and antibiotics, should be provided by government-sponsored programs to effectively and adequately manage measles-associated mortality. The immune amnesia associated with measles infection is a matter of general concern, especially in undernourished and immunocompromised individuals. Therefore, measles patients should be promptly evaluated for other infections and managed accordingly.

## Figures and Tables

**Table 1:** Progress towards measles elimination worldwide from 2000 to 2018 by the WHO.

WHO Region/Year (No. of Countries in Region)	% MCV1 Coverage	% Countries with ≥90% MCV1 Coverage	% MCV2 Coverage	% of Reporting Countries with <5 Measles Cases per 1 Million	No. of Reported Measles Cases	Measles Incidence per 1 Million	Estimated No. of Measles Cases (95% CI)	Estimated No. of Measles Deaths (95% CI)	Estimated % of Measles Mortality Reduction, 2000–2018	Cumulative No. of Measles Deaths Averted by Vaccination, 2000–2018
Africa										
2000 (46)	53	9	5	8	520,102	836	10,723,800 (7,718,000–17,119,100)	345,600 (236,300–562,100)	85	12,146,900
2018 (47)	74	30	26	47	125,426	118	1,759,000 (1,141,200–6,002,100)	52,600 (32,000–173,400)		
Americas										
2000 (35)	93	63	65	89	1754	2	8770 (4400–35,100)	NA	NA	97,100
2018 (35)	90	57	82	91	16,327	24	83,500 (41,800–334,200)			
Eastern Mediterranean										
2000 (21)	71	57	28	17	38,592	90	2,427,900 (1,503,800–3,892,900)	37,900 (21,700–64,000)	−29	2,820,600
2018 (21)	82	57	74	35	64,722	93	2,852,700 (2,293,700–4,265,200)	49,000 (36,700–72,500)		
Europe										
2000 (52)	91	62	48	45	37,421	50	860,176 (227,200–6,668,300)	400 (100–2200)	50	95,600
2018 (53)	95	89	91	34	82,523	98	861,800 (71,100–6,480,300)	200 (0–1800)		
South-East Asia										
2000 (10)	63	30	3	0	78,558	51	11,411,900 (8,764,600–15,572,100)	141,700 (100,100–199,600)	72	6,825,400
2018 (11)	89	82	80	36	34,741	18	3,803,800 (2,856,700–6,702,900)	39,100 (24,800–76,000)		
Western Pacific										
2000 (27)	85	48	2	30	177,052	105	2,786,500 (1,923,900–22,167,600)	10,000 (5200–74,200)	87	1,213,200
2018 (27)	95	59	91	77	29,497	15	408,400 (42,500–16,753,800)	1300 (100–2,786,500)		
Total										
2000 (191)	72	45	18	38	853,479	145	28,219,100 (20,141,900–65,455,000)	535,600 (363,400–901,700)	73	23,198,800
2018 (194)	86	61	69	54	353,236	49	9,769,400 (6,446,900–40,538,500)	142,300 (93,600–387,900)		

## Data Availability

Not applicable.
